# Transfer learning and vision transformer for the automatic diagnosis of cataracts in ophthalmological images

**DOI:** 10.3389/fdgth.2026.1805311

**Published:** 2026-06-30

**Authors:** Hugo Vega-Huerta, Camila Isabela Cuba-Aquino, Gari Mario Suca-Mariño, Ivan Adrianzén-Olano, Gisella Luisa Elena Maquen-Niño, Frida López-Córdova, Juan Carlos Lázaro-Guillermo, Gilberto Carrión-Barco, Katherin Vanessa Rodriguez-Zevallos, Denny John Fuentes-Adrianzén, Mario Chauca, Javier Elmer Cabrera-Díaz

**Affiliations:** 1 Universidad Nacional Mayor de San Marcos, Lima, Peru; 2 Universidad Nacional Toribio Rodríguez de Mendoza de Amazonas, Bagua, Peru; 3 Universidad Nacional Pedro Ruiz Gallo, Lambayeque, Peru; 4 Universidad Nacional Intercultural de la Amazonía, Ucayali, Peru; 5 Universidad Científica del Sur, Lima, Peru; 6 Universidad Ricardo Palma, Lima, Peru

**Keywords:** cataract, computer-assisted diagnosis, deep learning, fundus oculi, transfer learning

## Abstract

**Introduction:**

Cataracts continue to be the leading cause of preventable blindness worldwide and represent a significant public health challenge, particularly in rural and underserved regions where access to ophthalmology specialists and diagnostic infrastructure is limited. Early detection plays a crucial role in preventing visual impairment and improving treatment outcomes; however, large-scale screening programs are often constrained by the availability of trained professionals and specialized equipment. The purpose of this research was to develop and evaluate an automated cataract detection system based on deep learning using retinal fundus images in order to support early screening and improve accessibility to ophthalmological diagnosis.

**Methods:**

The proposed methodology followed an experimental quantitative approach that included dataset preparation, image preprocessing, model training, and performance evaluation. A labeled subset of 2,658 retinal fundus images extracted from the ODIR-5K dataset was used as the primary data source. The images underwent preprocessing procedures including normalization and noise reduction, followed by data augmentation techniques such as random rotations (±10°), scaling (90%–110%), and brightness and contrast adjustments. These transformations allowed the creation of a balanced dataset of 4,840 images, enhancing model generalization and reducing overfitting. Six deep neural network architectures were trained and evaluated: ResNet152, EfficientNet-v2S, Inception v3, MobileNet v3, DenseNet201, and Vision Transformer (ViT). Transfer learning with ImageNet pre-trained weights was applied together with selective fine-tuning of deeper layers and optimization using the Adam algorithm combined with a Cosine Annealing learning rate scheduler.

**Results:**

The results obtained indicate that ResNet152 is the bestperforming architecture with an accuracy of 99.10%, precision of 99.72%, sensitivity of 98.46%, and F1 score of 99.08%. It is concluded that deep convolutional neural network architectures, particularly ResNet152, provide highly effective performance for automated cataract detection from retinal fundus images.

**Discusion:**

The proposed system demonstrates strong potential as a clinical decision-support tool for large-scale screening programs, especially in resource-limited settings, as it can facilitate early diagnosis, improve access to ophthalmological care, and reduce the diagnostic workload of specialized medical personnel.

## 1 Introduction

Cataracts are the leading cause of preventable blindness worldwide, accounting for a large proportion of global vision loss (1). According to the World Health Organization (WHO), approximately 2.2 billion people are visually impaired or blind, of whom at least 1 billion have a visual impairment that could have been prevented or has not yet been treated, with cataracts accounting for more than 50% of these cases (2). In developing countries, early detection faces significant structural barriers due to a shortage of medical resources and limited infrastructure in rural areas. It is estimated that by 2050, the number of people with cataracts will reach 276 million, representing an unprecedented health challenge for global health systems (3).

While timely diagnosis is crucial for visual health, traditional methods (slit lamp, direct ophthalmoscopy) rely on highly trained specialists and expensive equipment. The diagnosis of cataracts through conventional clinical examination requires highly specialized professionals, whose availability is critically limited, with a ratio of 1.5 ophthalmologists per 100,000 inhabitants in low-income countries, compared to 10.6 per 100,000 in high-income countries (4).

In the last decade, artificial intelligence (AI), and specifically Deep Learning (DL), has demonstrated disruptive potential in medical image analysis, revolutionizing modern clinical practice (5). Artificial intelligence is defined as the ability of computer systems to perform tasks that traditionally require human intelligence, including pattern recognition, machine learning, and data-based decision-making (6), with machine learning being one of the most widely studied areas and applied to various human activities. Convolutional Neural Networks (CNN), deep learning architectures specialized in visual data processing using convolution layers that extract hierarchical features from images (7), have made it possible to automate the classification of eye diseases with levels of accuracy comparable to those of human experts, as evidenced in applications for retinopathy and other conditions (8). Transfer learning, a technique that allows pre-trained models to be reused on large datasets to adapt them to specific tasks with smaller datasets (9, 10), has emerged as a particularly effective strategy in AI-assisted medical diagnosis, significantly reducing data requirements and training time (11).

Recent research has validated the effectiveness of these technologies. A multicenter study demonstrated that CNN-based systems achieved 94.2% accuracy in cataract detection, with 92.8% sensitivity and 95.6% specificity, outperforming the performance of general ophthalmologists in triage settings (12). This background suggests that AI represents a robust solution for closing the ophthalmic care gap.

The practical contribution of this research is the development of an automated diagnostic system for the early detection of cataracts in ophthalmic images. This system, based on deep learning with convolutional neural networks (CNN) and transfer learning, aims to optimize the accuracy, accessibility, and efficiency of clinical diagnosis. Its impact is especially relevant in rural or low-income areas, where the availability of specialized ophthalmologists and advanced medical equipment is limited (13).

The technical proposal consists of integrating a clinical decision support system that automatically analyzes ophthalmological images. This software is designed to be incorporated into the medical workflow, facilitating efficient triage without requiring the immediate intervention of a specialist in the initial detection stages.

A central component of this proposal is the democratization of access to ophthalmological diagnosis. In rural areas, patients face significant barriers, such as geographical distances and a lack of medical infrastructure. This system mitigates these gaps by providing rapid, decentralized preliminary diagnosis, which would enable remote accessibility that would facilitate remote screening, significantly reducing the operating costs associated with traditional diagnosis, minimizing the need for unnecessary patient transfers, and optimizing the time of specialized medical staff for critical cases.

However, significant challenges remain in implementing these systems in real-world clinical settings. The diagnostic accuracy reported in controlled studies often decreases when the models are applied to populations with different demographic characteristics or variable image acquisition conditions (14). Furthermore, most current systems have not been specifically optimized to maximize sensitivity in triage settings, where the detection of positive cases is a priority to prevent patients with cataracts from not being referred for specialized evaluation.

Given this problem, the following question arises: To what extent can a Transfer Learning-based system with CNN architecture optimize the level of accuracy in the diagnosis of cataracts from ophthalmological images in rural areas? Consequently, the main objective of this study was to develop and evaluate an automated system using Transfer Learning that allows for efficient classification of fundus images, specifically seeking to maximize diagnostic sensitivity and accuracy to avoid false negatives in triage stages.

The main contributions of this study are fourfold. First, a systematic and controlled comparison of six neural network models that include a transfer learning or Vision Transformer model as their backbone: ResNet152, EfficientNet-v2S, Inception v3, MobileNet v3, DenseNet201, and Vision Transformer (ViT-B/16), evaluated under identical experimental conditions (same dataset, preprocessing pipeline, augmentation strategy, optimizer, and learning rate scheduler) for binary cataract detection from retinal fundus images. Second, a state-of-the-art classification performance (99.10% accuracy, 99.72% precision, 98.46% sensitivity, F1-score of 99.08%) using a moderate-sized, publicly available dataset (ODIR-5K subset, *n* = 2,658 images; 4,840 after augmentation), without proprietary large-scale data, showing that architectural optimization can compensate for the absence of massive datasets. Third, we implement a training protocol combining selective layer freezing with Cosine Annealing scheduling, strategic dropout regularization, and early stopping, explicitly designed to maximize sensitivity in triage settings where false negatives carry the greatest clinical cost. The selective fine-tuning strategy was applied consistently across all six neural network models, with each pre-trained backbone frozen except for its deepest representational block. This uniform freezing strategy ensured that pre-trained ImageNet representations were preserved in earlier layers, which encode general visual features transferable across domains, while allowing task-specific adaptation exclusively in the high-level semantic layers most relevant to fundus image classification. Fourth, a external validation on a large heterogeneous dataset of 92,856 images (15), corroborating the model's generalizability beyond the training distribution and its potential for real-world clinical deployment, particularly in resource-limited settings where access to specialized ophthalmological services is critically constrained.

## 2 Background

In recent years, deep learning models, specifically convolutional neural networks (CNNs), have proven to be highly effective tools in medical image analysis, enabling an accurate approach to detecting cataracts and other ophthalmic pathologies (16). The integration of attention mechanisms and transformer architectures has revolutionized medical image analysis, allowing models to capture long-range dependencies and contextual features that traditional CNNs cannot efficiently model. These networks, which rely on the ability of machines to learn complex patterns from large volumes of data, have been widely used to overcome the limitations of traditional diagnostic methods (17). The ability of CNNs to process large amounts of image data has been particularly useful in contexts where access to specialized equipment is limited, as is the case in many rural areas of Peru.

The use of CNNs in ophthalmology, particularly in the classification of fundus images, has enabled significant advances in the early detection of cataracts, in many cases surpassing diagnoses made by human experts. Among the most common approaches, EfficientNet, ResNet, and Inception have been widely used to improve diagnostic accuracy. Multi-class classification of retinal diseases from ophthalmoscopy images has also been addressed through Vision Transformer architectures, with ViT-L/16 achieving 98.1% accuracy on a balanced dataset of 4,217 images, outperforming CNN-based counterparts in multi-disease classification tasks (18). Similarly, multi-disease retinal diagnosis from ultra-wide-field fundus images has been explored using ResNet152, Vision Transformer, and related architectures over a dataset of 4,697 images, with ResNet152 achieving the best performance at 89.17% accuracy and an AUC of 96.47% (19).

Data Augmentation and K-Fold techniques have been instrumental in improving model robustness and reducing the risk of overfitting, allowing systems to be more general and applicable to diverse clinical settings. These techniques are essential when the available data is limited or unbalanced, as is the case in many health institutions in rural areas of Peru (20, 21). Fundus image classification into three categories using ResNet-50 and Inception V3 with transfer learning further demonstrated that pretrained architectures with fine-tuning can yield competitive results 93.81% and 91.76% accuracy, respectively—even with reduced dataset sizes (22).

The automated detection of specific cataract subtypes has also been explored. A deep learning algorithm based on a lens shadow projection theory applied to ultra-wide-field fundus images achieved 80% accuracy and 88.2% sensitivity for high-risk posterior polar cataract screening, validated on 103 clinical cases (23). Likewise, lightweight architectures such as MobileNet have proven effective not only for cataract-related tasks but also for closely related ophthalmic conditions such as glaucoma, confirming their suitability for clinical deployment in resource-constrained environments across multiple public reference datasets (24).

Currently, various deep learning models have shown promising results in the classification and detection of cataracts, highlighting the effectiveness of ensemble methods and multi-architecture comparisons (25). Lightweight frameworks such as CSDNet have demonstrated the ability to operate efficiently on devices with limited memory and storage, significantly reducing trainable parameters without compromising representational capacity (26). These advances underscore the disruptive potential of AI to automate ophthalmic screening, optimizing time and accessibility—a critical factor in regions with poor healthcare infrastructure—while the robustness and generalization capacity of these models have been reinforced through data augmentation and transfer learning techniques, essential strategies for avoiding overfitting in limited datasets.

The model proposed in this paper, based on an optimized convolutional neural network, draws inspiration from these advances to develop an accessible solution that facilitates the early detection of cataracts in rural areas of Peru, where access to specialized equipment is limited (27). The combination of data augmentation and the integration of low-quality images will be essential to ensure the viability of this model in contexts with limited infrastructure, reinforcing the importance of these technologies in improving access to healthcare and offering faster and more accessible diagnoses in underserved communities (28).

Table 1 summarizes the performance metrics of various previous studies analyzed, of CNN-based models for cataract detection, using the metrics of Accuracy (ACC), Precision (PREC), and Sensitivity (SEN), which validates the choice of similar approaches for research, adapted to the local clinical context and with limited access technologies.

**Table 1:** Summary of related studies on cataract detection.

Article Title	Data Set	Architecture	Method	ACC %	PREC %	SEN %	Quartil
Cataract detection based on ocular B-ultrasound images by collaborative monitoring deep learning (17)	15,943 ocular ultrasound images (B-ultrasound)	YOLO-v3 + DenseNet-161 + Fourier Descriptors + GLCM	Collaborative Monitoring Deep Learning) with extraction of depth, shape, and texture features	98	–	–	Q1
Fundus Photograph-Based Cataract Evaluation Network Using Deep Learning (29)	Beijing Eye Study 2011 (1,340 images)	Dual-Stream Cataract Evaluation Network based on ResNet50	Inclusion of Metadata, Ensemble	97.62	–	–	Q2
Universal artificial intelligence platform for collaborative management of cataracts (30)	CMAAI Chinese Medical Alliance for Artificial Intelligence (37,638 images)	ResNet (CNN)	Three-step labeling: (1) capture mode recognition, (2) cataract diagnosis, (3) referable cataract detection	>99 (AUC 99.82–99.96)	–	–	Q1
Cataract and glaucoma detection based on Transfer Learning using MobileNet (21)	Kaggle: (300 normal images, 100 cataract images, 100 glaucoma images)	MobileNetV1 and MobileNetV2	Transfer Learning with depth-wise separable convolutions	99 (cataract), 98 (glaucoma),	93 (cataract), 76 (glaucoma),	93 (cataract), 72 (glaucoma),	Q1
Multi-class classification of retinal eye diseases using transfer learning-based vision transformers (18)	Ophthalmoscopy images (4,217 images)	ViT-L/16 (AugReg), ResNet50, DenseNet121, Inception-ResNetV2 + 6 ViT variants	Transfer Learning, Data Augmentation, Regularization	98.1	–	–	Q1
Innovative utilization of ultra-wide field fundus images and deep learning algorithms for screening high-risk posterior polar cataract (23)	Ultra-wide-field fundus images (546 training, 103 validation)	CNN (optimized architecture)	Shadow projection theory, Data Augmentation	80	–	88.2	Q1
Fundus image classification using Inception V3 and ResNet-50 for the early diagnostics of fundus diseases (22)	Fundus images (∼200 images/class, 3 classes)	ResNet-50, Inception V3	Transfer Learning, Hyperparameter tuning	93.81 (ResNet-50), 91.76 (Inception V3)	–	–	Q2
Retinal disease diagnosis using deep learning on ultra-wide-field fundus images (19)	Ultra-wide-field fundus images (4,697 images)	ResNet152, ViT, InceptionResNetV2, RegNet, ConVNext	Transfer Learning, Brightness/Contrast Enhancement, Data Augmentation	89.17	–	–	Q1
Identification of glaucoma from retinal fundus images using deep learning model, MobileNet (24)	9 public datasets (DrishtiGS, REFUGE, EyePACS AIROGS-Light, BEH, PAPILA, among others)	MobileNetV2	Transfer Learning, depth-wise separable convolutions	–	–	–	Q3

In this context, the present research work compared and evaluated the most current and representative versions of transfer learning architectures, specifically ResNet152, EfficientNet v2_s, Inception v3, MobileNet v3, and DenseNet201, which were used as headers of the same convolutional network (CNN). This strategy allowed for an objective analysis of the impact of each architecture on model performance, demonstrating that the use of transfer learning contributes to a reduction in the number of epochs required for convergence, a reduction in processing time, and a substantial improvement in performance accuracy and stability, especially in scenarios with limited datasets (31). The results obtained support the effectiveness of the proposed approach and its relevance for biomedical applications and highly complex image classification.

## 3 Methods

The methodology adopted follows an experimental quantitative approach and is structured in five sequential phases aligned with the system design: data acquisition, preprocessing, CNN model training, performance evaluation, and interface implementation.

This methodological structure aligns with approaches validated in prior ophthalmic deep learning research. Vijayaraghavan (32) applied the CRISP-DM framework for detecting multiple ocular diseases including cataract using transfer learning techniques such as VGG-19, establishing a systematic pipeline from preprocessing through model evaluation. This procedural reference closely mirrors the sequential phases adopted in the present study, enabling direct comparison of results under analogous experimental conditions.

**Figure 1:**
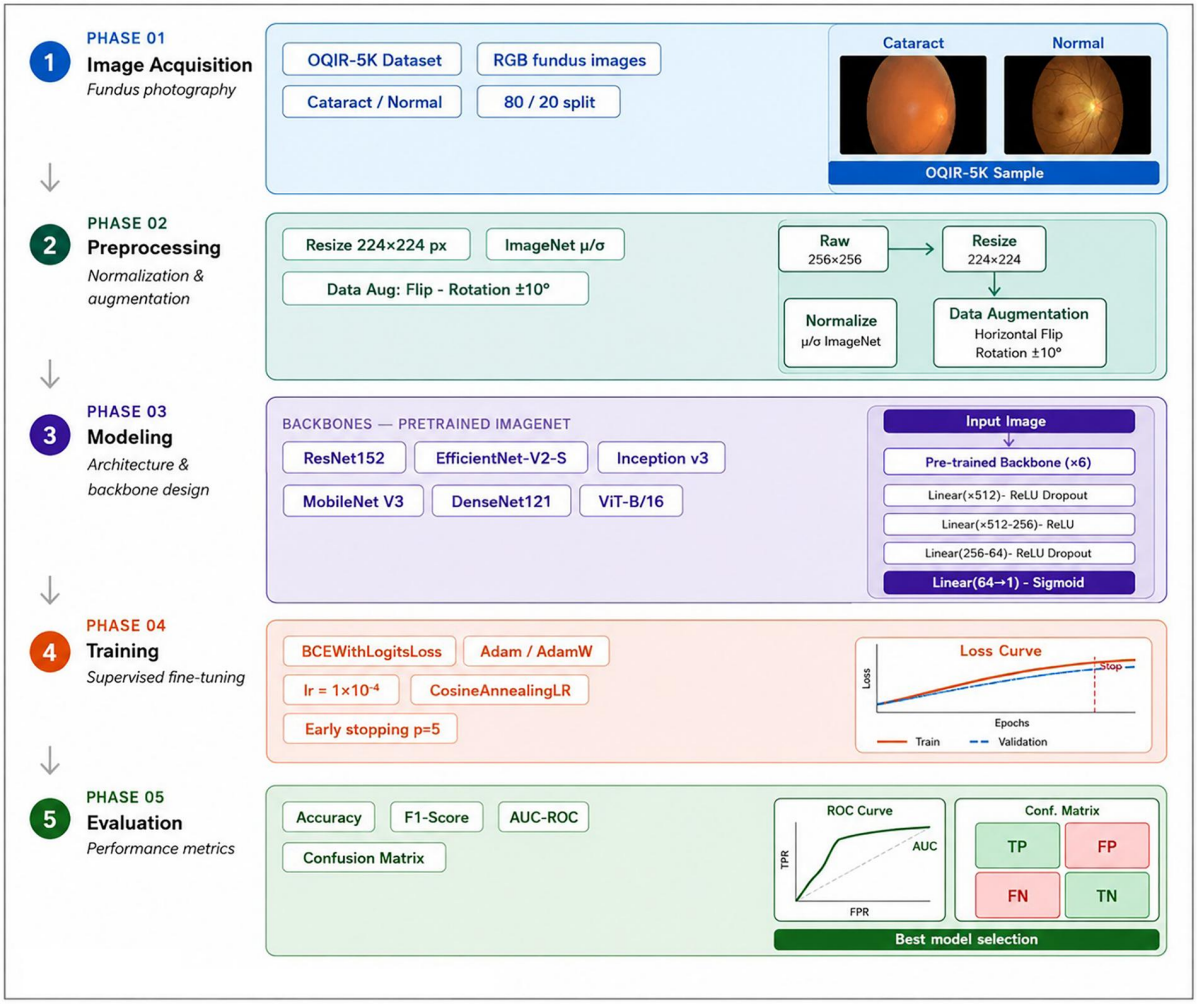
Process of the proposed system development methodology.

This methodology is visualized in a flowchart, presented in Figure 1, which illustrates the interaction of five key components of the system:

### 3.1 Image acquisition

To create the dataset for this study, the ODIR-5K (Ocular Disease Intelligent Recognition) dataset was selected, a publicly available structured ophthalmic database compiled by Shanggong Medical Technology Co., Ltd. from real clinical records collected across multiple hospitals and medical centers in China (33). This dataset was released for the 2019 Peking University International Competition on Ocular Disease Intelligent Recognition (ODIR-2019), hosted on the Grand Challenge platform (https://odir2019.grand-challenge.org/dataset/). The dataset comprises color fundus photographs, providing paired left- and right-eye images along with physician-assigned diagnostic keywords, classifying each patient into one or more of eight diagnostic categories: Normal (N), Diabetes (D), Glaucoma (G), Cataract (C), Age-related Macular Degeneration (A), Hypertension (H), Myopia (M), and other diseases/abnormalities (O). Images were captured in clinical settings using commercial fundus cameras from Canon, Zeiss, and Kowa, resulting in variable image resolutions across institutions. The complete dataset comprises 8,000 images distributed into 7,000 for training and 1,000 for testing, corresponding to 5,000 patients.

This dataset is also hosted in a publicly available repository on Kaggle (34), and was accessed for this research The inclusion criteria were as follows:

1. Quality and Resolution: The dataset consists of images with a minimum resolution of 224 × 224 pixels.
2. Clinical Validation: The dataset contains images labeled by specialist ophthalmologists.

**Figure 2:**
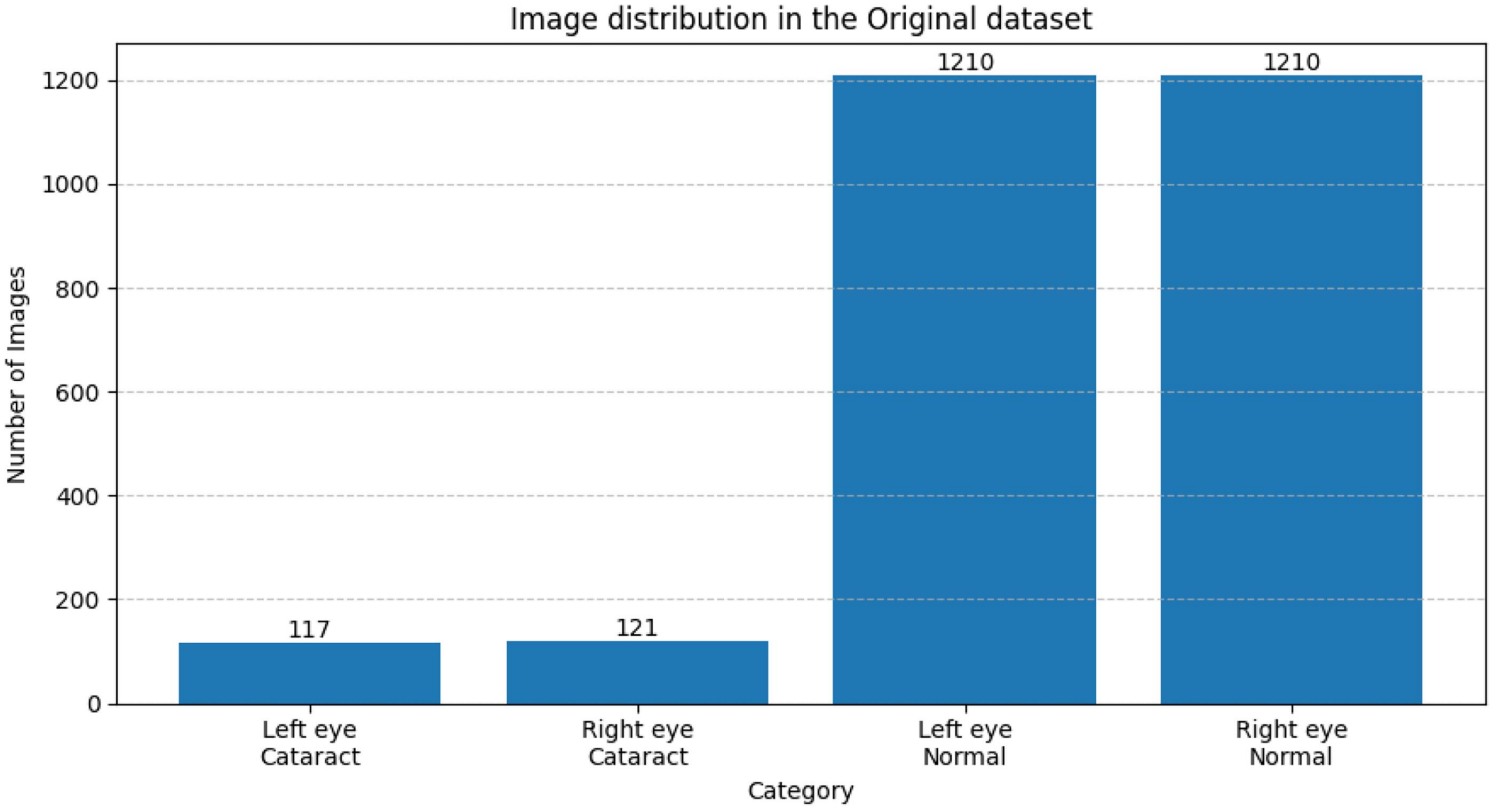
Distribution of images by class (cataract vs. Normal).

As this research only sought to determine the relationship between the presence or absence of cataracts, only 2,658 images corresponding to the Normal (N) and Cataract (C) categories were selected. Figure 2 shows the distribution of images within the two categories, verifying that there is a class imbalance (91% of images for the normal category vs. 9% of images for the cataract category), a condition that was subsequently addressed using data augmentation techniques.

**Figure 3:**
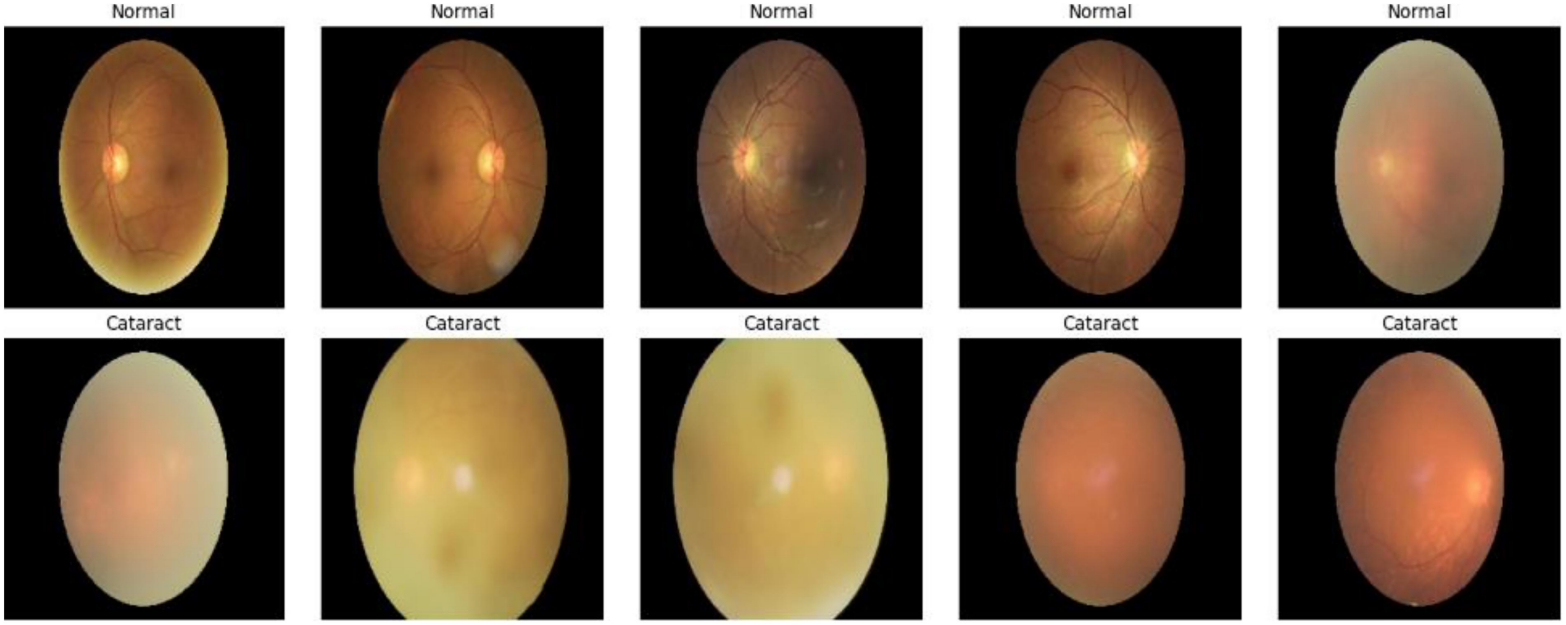
Sample images for each class label (cataract vs. Normal).

Figure 3 presents representative sample images from the dataset used in this study, illustrating the two class labels considered: Normal and Cataract. The first row shows examples of normal retinal fundus images with clear retinal structures, while the second row displays images corresponding to cataract cases, where lens opacity leads to reduced contrast and blurred retinal features. These examples highlight the visual differences between the two classes and provide an overview of the type of images used to train and evaluate the deep learning models.

### 3.2 Image preprocessing

The objective of this phase was to standardize the inputs for the neural network and mitigate the class imbalance in the dataset. The Keras ImageDataGenerator class was used to implement the following processes:

Noise Reduction: Gaussian filters were applied to smooth visual artifacts while preserving relevant diagnostic details.

Data Balancing: To address the class imbalance problem and improve generalization, a data augmentation strategy was implemented using the ImageDataGenerator framework. This approach allowed realistic variations of the original images to be generated, preserving relevant clinical features and minimizing the risk of introducing artificial artifacts. In particular, controlled transformations were applied, including:

1. Random rotations of up to 10 degrees (rotation_range = 10).
2. Zoom variations of up to 10% (zoom_range = 0.1).
3. Brightness adjustments within the range of 0.9–1.1, thus simulating the common variability present during the image acquisition process [brightness_range = (0.9, 1.1)].
4. Constant fill mode with a padding value equal to zero (cval = 0), ensuring uniform background treatment after geometric transformations (fill_mode = “constant”, cval = 0).
5. Conversion to tensor using ToTensor, transforming PIL format images [range (0, 255)] to PyTorch tensors [range (0, 1)].

**Figure 4:**
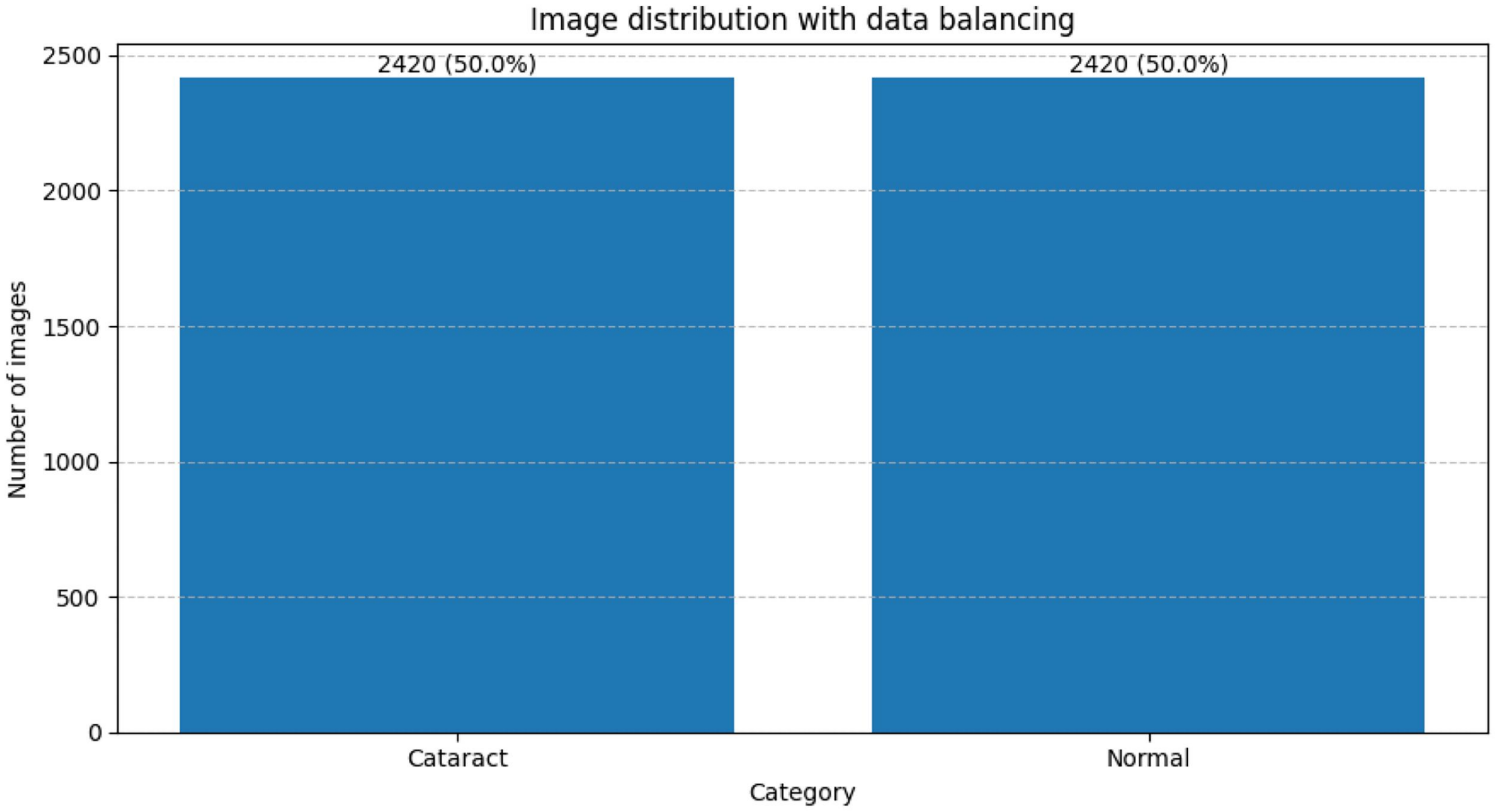
Distribution of images with data balancing by class (cataract Vs. Normal).

As a result of the augmentation strategy, 2,182 additional images of the cataract class were generated, reaching a total of 2,420 images in that class, as shown in Figure 4. This allowed the dataset to be balanced with respect to the normal class. Overall, this strategy contributed to greater stability during training, reduced overfitting, and a significant improvement in the performance of the evaluated models.

**Figure 5:**
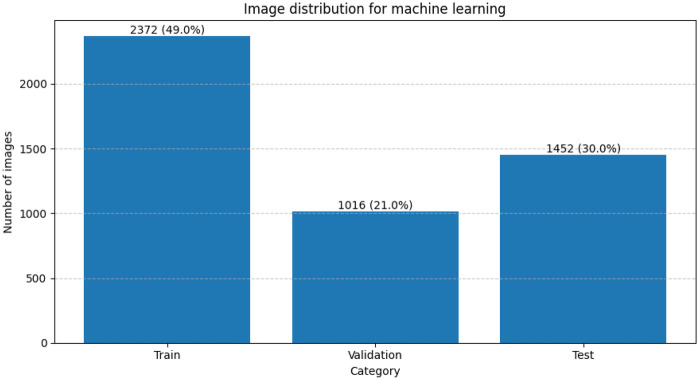
Distribution of images by ML process (train, validation, and test).

Subsequently, the dataset was partitioned into three subsets: training (49%), validation (21%), and evaluation (30%), as illustrated in Figure 5. This partition was organized using PyTorch's ImageFolder directory structure, where each subset was stored in separate paths with subdirectories per class.

The PyTorch libraries were used together with the torchvision module to implement the following processes:

#### 3.2.1 Normalization and resizing

All images were resized to a resolution of 224 × 224 pixels using the Resize transformation in order to homogenize the input dimensions required by the architectures considered, with the exception of the Inception architecture, which required a size of 299 × 299 pixels. Subsequently, the intensity values of each image were normalized using the Normalize transformation with precomputed statistical parameters from the ImageNet dataset (mean = [0.485, 0.456, 0.406] and standard deviation = [0.229, 0.224, 0.225]), which standardizes the distribution of pixel values to the approximate range of [−2.5, 2.5] in each RGB channel. This normalization helps facilitate the convergence of the optimization algorithm during training and ensures compatibility with the pre-trained weights in ImageNet.

#### 3.2.2 Data loader configuration

To optimize the training and evaluation process, specific data loaders (DataLoader) were implemented for each set, with the following configurations:

1. Training Set (train_loader): Configured with batch_size = 32 to process 32 images simultaneously in each iteration. Shuffle = True was enabled to randomize the order of samples in each epoch, thus reducing the risk of learning spurious patterns associated with the order of presentation. Num_workers = 4 was used for parallel data loading, taking advantage of multiple processor cores, and pin_memory = True was used to accelerate data transfer to the GPU using pinned memory.
2. Validation and Test Sets (val_loader and test_loader): These were configured with batch_size = 32, but with shuffle = False to maintain a consistent and reproducible order during evaluation. num_workers = 4 and pin_memory = True were maintained to optimize loading times, which is especially relevant in large test sets.

This data loader configuration ensured an efficient flow of information from storage to the GPU, minimizing wait times (I/O bottleneck) and maximizing the use of computational resources throughout the training and evaluation process.

### 3.3 Modeling

In this stage, six deep learning models were developed for supervised learning, five based on a transfer learning (TL) approach and one with Vision Transformer (ViT). The models used six base architectures: ResNet152, EfficientNet-V2-S, Inception v3, MobileNet v3, DenseNet 201, and Vit Transformer, all pre-trained on the ImageNet dataset (IMAGENET1K_V1). The selection of architectures with Transfer Learning is based on their ability to extract high-level hierarchical representations using deep convolutional blocks, which mitigate the problem of gradient vanishing and allow for the training of deep networks with greater stability and performance.

Each of the six architectures selected for this study has distinct theoretical foundations and documented relevance in medical image analysis tasks. ResNet152 (19) is a 152-layer residual network whose skip connections address the vanishing gradient problem, enabling stable training of very deep networks. Its depth allows the extraction of highly abstract hierarchical features, and its performance in ophthalmic image classification has been extensively validated in the literature (8, 29). EfficientNet-V2-S (23) introduces compound scaling of depth, width, and resolution, achieving superior accuracy-to-computational-cost ratios. Its Fused-MBConv blocks and progressive learning strategy make it particularly efficient when labeled data is limited, a common scenario in clinical datasets. Inception v3 (22) employs parallel multi-scale convolutional modules that simultaneously capture features at different receptive field sizes, making it well suited to detect heterogeneous visual patterns such as the varying degrees of lens opacity present in cataract images. Its auxiliary classifier additionally mitigates gradient vanishing during training. MobileNet V3 (24) is a lightweight architecture designed for efficient deployment, combining inverted residual blocks with Squeeze-and-Excitation modules and hard-swish activations. Although its parameter count is substantially lower than deeper counterparts, it has demonstrated competitive accuracy in retinal image classification (21), making it relevant for resource-constrained clinical environments. DenseNet201 (18) connects each layer to all subsequent layers, maximizing feature reuse and enabling strong gradient flow with fewer parameters. This dense connectivity facilitates the preservation of fine-grained texture details, which are diagnostically relevant in fundus images. Finally, Vision Transformer ViT-B/16 (18) departs from the convolutional paradigm by partitioning the image into 16 × 16 patches and modeling their global relationships through multi-head self-attention mechanisms. This capacity to capture long-range dependencies across the entire image field complements the local feature extraction of CNNs and has shown growing relevance in medical imaging tasks where global contextual understanding is critical (35). The inclusion of ViT alongside the five CNN-based architectures enables a direct empirical comparison between convolutional and attention-based approaches for cataract detection.

#### 3.3.1 Neural network architecture

The input images were resized to 224 × 224 pixels, in accordance with the requirements of the pre-trained architectures, with the exception of Inception_v3, which required images of 299 × 299 pixels. During the fine-tuning process, a selective layer freezing strategy was applied consistently across all six neural network models: the parameters of the initial layers were frozen and only the weights of the deepest representational block of each backbone were updated, allowing task-specific adaptation to the fundus image domain without compromising the general visual representations learned from ImageNet. Specifically, in ResNet152, all layers were frozen except layer4 (the fourth and deepest residual block, comprising three bottleneck units); in EfficientNet-V2-S, all layers were frozen except the last convolutional block (features [−1]); in Inception v3, all layers were frozen except the Mixed_7c module (the deepest inception block); in MobileNet v3, all layers were frozen except the last inverted residual block (features [−1]); in DenseNet201, all layers were frozen except denseblock4 and its associated batch normalization layer norm5; and in the Vision Transformer ViT-B/16, all encoder layers were frozen except the two deepest Transformer encoder layers (encoder.layers.10 and encoder.layers.11), which correspond to the last two of twelve self-attention blocks. This strategy favors the progressive specialization of each model in patterns relevant to the clinical dataset, while reducing the risk of overfitting and preserving the low- and mid-level visual representations, such as edge detectors and texture filters.

The original fully connected layer of each base architecture (ResNet152, EfficientNet-V2-S, Inception v3, MobileNet v3, DenseNet 121, and Vit Transformer) was replaced by a custom neural network oriented towards a binary classification task, composed of a sequence of linear layers with dimensions 512, 256, and 64 neurons, each followed by ReLU activation functions. Two Dropout layers with a rate of 0.5 were strategically positioned after the 512 and 64 neuron layers, with the purpose of improving generalization capacity through the random deactivation of neurons during training. Finally, the output layer consists of a neuron implemented using the BCEWithLogitsLoss loss function, which numerically stably integrates the sigmoid function and binary cross-entropy, thus avoiding numerical stability problems associated with the sequential application of these operations, as shown in Table 2.

**Table 2:** Neural network architecture with base model.

Layer (type:depth-idx)	Ouput Shape
Base Model (TL or ViT)	[1, n_features]
─Sequential: 1–3	[1, 1]
└─Linear: 2–4	[1, 512]
└─ReLU: 2–5	[1, 512]
└─Dropout: 2–6	[1, 512]
└─Linear: 2–7	[1, 256]
└─ReLU: 2–8	[1, 256]
└─Linear: 2–9	[1, 64]
└─ReLU: 2–10	[1, 64]
└─Dropout: 2–11	[1, 64]
└─Linear: 2–12	[1, 1]

Table 3 summarizes the main architectural and training features of the six models evaluated. All were initialized with ImageNet pretrained weights and adapted for binary cataract classification through a uniform custom head of four fully connected layers with ReLU activations and Dropout (*p* = 0.5). Fine-tuning was selectively applied to the deepest convolutional or attention block of each backbone—layer4 for ResNet152, features[−1] for EfficientNet-V2-S and MobileNet v3, Mixed_7c for Inception v3, denseblock4 + norm5 for DenseNet201, and encoder.layers.10–11 for ViT-B/16—while all remaining layers were kept frozen.

**Table 3:** Main features of the six models.

Indicator	ResNet152	EfficientNet-V2-S	Inception v3	MobileNet V3 (Large)	DenseNet201 (art. cita 121)	ViT-B/16
Input Dimension	224 × 224 × 3 (1, 3, 224, 224)	224 × 224 × 3 (1, 3, 224, 224)	299 × 299 × 3 (1, 3, 299, 299)	224 × 224 × 3 (1, 3, 224, 224)	224 × 224 × 3 (1, 3, 224, 224)	224 × 224 × 3 (1, 3, 224, 224)
Output Dim. Base Model	[1, 2,048, 7, 7]	[1, 1,280, 7, 7]	[1, 192, 8, 8]	[1, 960, 7, 7]	[1, 1,920, 7, 7]	[1, 197, 768]
	→ AvgPool	→ AvgPool	→ AvgPool + Dropout	→ AvgPool	→ AvgPool	→ CLS token
	→ [1, 2,048,1,1]	→ [1, 1,280,1,1]	→[1, 2,048, 1,1]	→ [1, 960,1,1]	→ [1, 1,920]	→ [1, 768]
Layers	Linear (2,048 → 512) + ReLU + Dropout (0.5) Linear (512 → 256) + ReLU Linear (256 → 64) + ReLU + Dropout (0.5) Linear (64 → 1)	Linear (1,280 → 512) + ReLU + Dropout (0.5) Linear (512 → 256) + ReLU Linear (256 → 64) + ReLU + Dropout (0.5) Linear (64 → 1)	Linear (2,048 → 512) + ReLU + Dropout (0.5) Linear (512 → 256) + ReLU Linear (256 → 64) + ReLU + Dropout (0.5) Linear (64 → 1)	Linear (960 → 512) + ReLU + Dropout (0.5) Linear (512 → 256) + ReLU Linear (256 → 64) + ReLU + Dropout (0.5) Linear (64 → 1)	Linear (1,920 → 512) + ReLU + Dropout (0.5) Linear (512 → 256) + ReLU Linear (256 → 64) + ReLU + Dropout (0.5) Linear (64 → 1)	Linear (768 → 512) + ReLU + Dropout (0.5) Linear (512 → 256) + ReLU Linear (256 → 64) + ReLU + (0.5) Linear (64 → 1)
Fine-tuning	lr = 1 × 10^−4^	lr = 1 × 10^−4^	lr = 1 × 10^−4^	lr = 1 × 10^−4^	lr = 1 × 10^−4^	lr = 1 × 10^−4^
	Scheduler:	Scheduler:	Scheduler:	Scheduler:	Scheduler:	weight_decay
	CosineAnn.	CosineAnn.	CosineAnn.	CosineAnn.	CosineAnn.	= 1 × 10^−4^
	T_max = 10,	T_max = 10,	T_max = 10,	T_max=10,	T_max=10,	Scheduler: CosineAnn.
	*η*_min = 1 × 10^−6^	*η*_min = 1 × 10^−6^	*η*_min = 1 × 10^−6^	*η*_min = 1 × 10^−6^	*η*_min=1 × 10^−6^	T_max=10, *η*_min=1 × 10^−6^
	Dropout: *p* = 0.5 (×2)	Dropout: *p* = 0.5 (×2)	Dropout: *p* = 0.5 (×2)	Dropout: *p* = 0.5 (×2)	Dropout: *p* = 0.5 (×2)	Dropout: *p* = 0.5 (×2)
	Batch size: 32	Batch size: 32	Batch size: 32	Batch size: 32	Batch size: 32	Batch size: 32
	Épocas máx.: 20	Épocas máx.: 20	Épocas máx.: 20	Épocas máx.: 20	Épocas máx.: 20	Épocas máx.: 20
	Early stopping: *p* = 5	Early stopping: *p* = 5	Early stopping: *p* = 5	Early stopping: *p* = 5	Early stopping: *p* = 5	Early
	Aum.: HFlip, Rot (10°)	Aum.: HFlip, Rot (10°)	Aum.: HFlip, Rot (10°)	Aum.: HFlip, Rot (10°)	Aum.: HFlip, Rot (10°)	stopping: *p* = 5
						Aum.: HFlip, Rot (10°)
Activation Function	Cabecera: ReLU	Cabecera: ReLU	Cabecera: ReLU	Cabecera: ReLU	Cabecera: ReLU	Cabecera: ReLU
	Salida: Sigmoide	Salida: Sigmoide	Salida: Sigmoide	Salida: Sigmoide	Salida: Sigmoide	
	(implícita BCE)	(implícita BCE)	(implícita BCE)	(implícita BCE)	(implícita BCE)	

### 3.4 Training

A consistent training protocol was shared across models: Loss Function was BCEWithLogitsLoss, Adam (lr = 1 × 10^−4^), CosineAnnealingLR, batch size 32, 20 epochs maximum, and early stopping (patience = 5). ViT-B/16 uniquely employed AdamW with weight decay of 1 × 10^−4^.

The six models were trained for 20 epochs with a batch size of 32, to process 32 images simultaneously in each iteration. The Adam optimizer was used with an initial learning rate of 1 × 10^−4^, applied only to parameters with enabled gradients, thus ensuring selective updating of the weights of the last convolutional block and the custom neural network. Cosine Annealing learning rate scheduler (CosineAnnealingLR) was implemented with a period ofT_max_ = 10 s epochs and a minimum rate of 1 × 10^−6^, allowing for a progressive and non-linear decrease in the learning rate, favoring more stable convergence and improving generalization capacity. To mitigate overfitting, an Early Stopping mechanism was incorporated with a patience of 5 epochs, stopping training when the validation loss showed no significant improvement. Likewise, a checkpointing system was implemented, which preserved the model with the best performance in terms of validation loss, storing both the model's weights and optimizer state to allow training to resume if necessary.

The neural network was developed and implemented in Python, using the PyTorch library and the torchvision module to load the pre-trained architecture. The torchinfo library was also used to visualize the model architecture. Training was performed with GPU acceleration (CUDA), using a graphics card, optimizing processing times and enabling efficient handling of deep networks. The implementation of the progress bar using the tqdm library facilitated real-time monitoring of the training process.

### 3.5 Evaluation and diagnosis

The performance of the models was evaluated on the independent test set (30% of the total dataset), previously separated and not used during training or validation. The evaluation process was performed by setting the model to inference mode using model.eval(), which disables the Dropout layers and fixes the behavior of the batch normalization layers, ensuring deterministic and reproducible predictions.

During the evaluation phase, the images in the test set were processed using test_loader, applying the previously defined deterministic transformations (resizing to 224 × 224 pixels, conversion to tensor, and normalization with ImageNet statistics). For each batch of 32 images, the model generated logits that were converted to probabilities using the sigmoid function, applying a decision threshold of 0.5 for binary classification (cataract vs. normal). This process was performed under the torch.no_grad() context to disable gradient calculation, thus optimizing GPU memory usage and accelerating inference time.

The evaluation was performed by iteratively going through the entire test set, accumulating performance metrics in each batch. For each processed image, the binary prediction (obtained after applying the threshold of 0.5 to the probabilities) was compared with the true label, counting correct predictions using a test_correct counter. Simultaneously, all true labels and predictions were stored in lists (y_true, y_pred), which were then transferred from the GPU to the CPU using.cpu().numpy() and converted to one-dimensional NumPy arrays using.ravel(), facilitating their processing with scientific analysis libraries. The BCEWithLogitsLoss loss function was computed for each batch during evaluation, accumulating in test_loss. At the end of the complete run of the test set, this accumulated loss was averaged by dividing it by the total number of batches (len(test_loader)), providing an aggregate measure of classification error.

Standard performance metrics were calculated using the y_true and y_pred lists as input values using the scikit-learn library: Accuracy (proportion of correct predictions out of the total number of samples evaluated), precision (proportion of true positives out of the total positive predictions, quantifying the reliability of cataract detections), sensitivity—Recall (the model's ability to correctly identify positive cases of cataracts, true positive rate, a critical metric in medical applications where early detection is essential), specificity (proportion of negative cases correctly classified, true negative rate), f1-score (harmonic mean between precision and sensitivity, providing a balanced metric that is particularly useful in scenarios with potentially unbalanced classes).

Table 4 shows the classification reports generated by each base architecture using the classification_report() function from scikit-learn, which presents the metrics of precision, sensitivity, F1-score, and support for each class (“normal” and “cataract”), as well as macro and weighted averages.

**Table 4:** Classification report summarizing the evaluation metrics of the models.

Class	Metric	ResNet152	EfficientNet v2S	Inception v3	Mobile v3	DenseNet 121	ViT B/16
Normal	Precision	0.99	0.96	0.97	0.95	0.98	0.97
	Recall	1.00	0.99	1.00	1.00	1.00	1.00
	F1-Score	0.99	0.97	0.98	0.97	0.99	0.98
	Support	740	740	740	740	740	740
Cataract	Precision	1.00	0.99	1.00	1.00	1.00	1.00
	Recall	0.98	0.96	0.97	0.95	0.98	0.97
	F1-Score	0.99	0.97	0.98	0.97	0.99	0.98
	Support	712	712	712	712	712	712
Macro avg	Precision	0.99	0.97	0.98	0.97	0.99	0.98
	Recall	0.99	0.97	0.98	0.97	0.99	0.98
	F1-Score	0.99	0.97	0.98	0.97	0.99	0.98
	Support	1,452	1,452	1,452	1,452	1,452	1,452
Weighted avg	Precision	0.99	0.97	0.98	0.97	0.99	0.98
	Recall	0.99	0.97	0.98	0.97	0.99	0.98
	F1-Score	0.99	0.97	0.98	0.97	0.99	0.98
	Support	1,452	1,452	1,452	1,452	1,452	1,452

**Figure 6:**
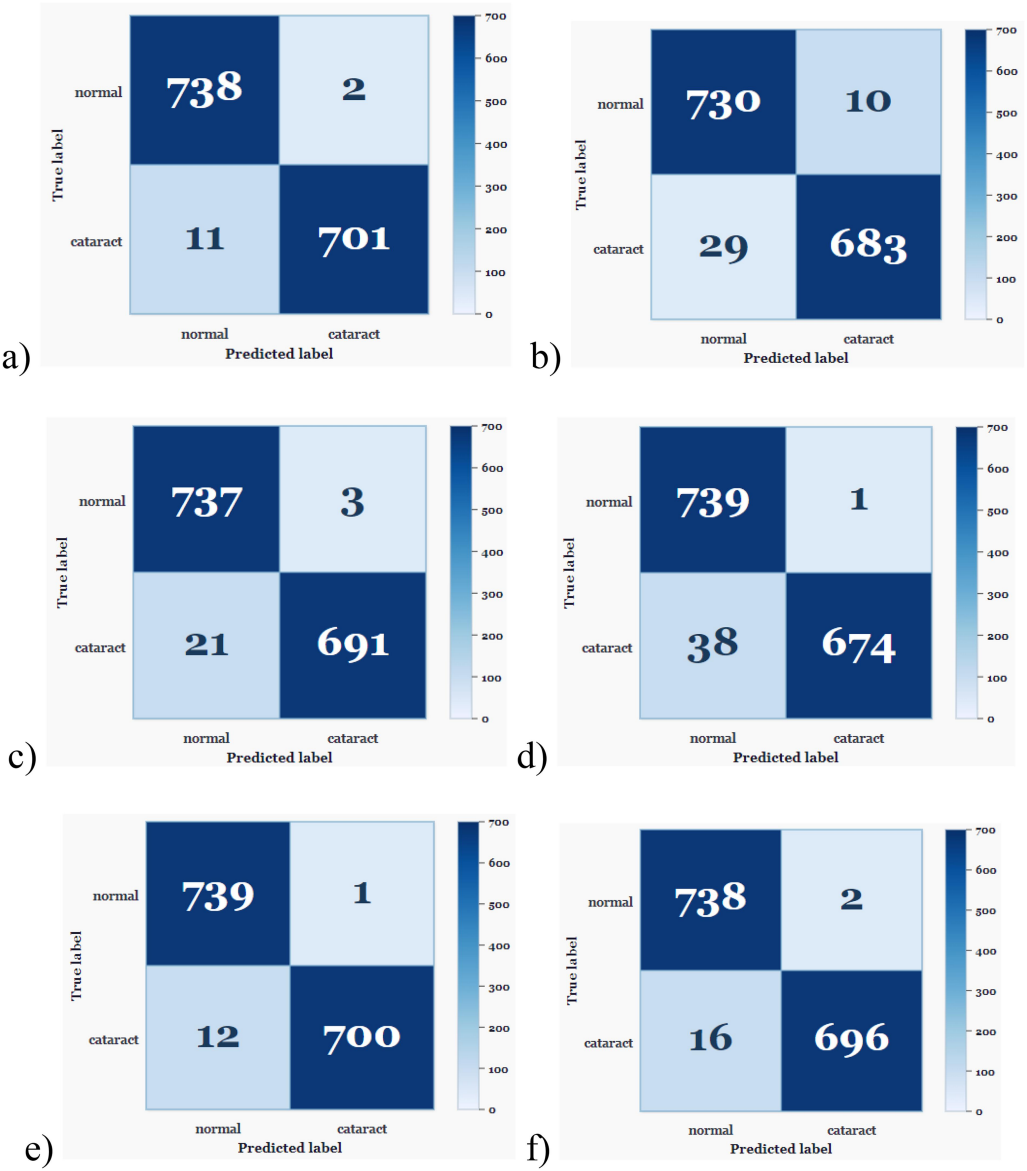
Confusion matrix **(a)** ResNet152, **(b)** efficientNet v2S, **(c)** inception v3, **(d)** Mobile v3, **(e)** DenseNet201, and **(f)** vision transformer ViT B/16.

Additionally, the confusion matrix was constructed using confusion_matrix() as shown in Figure 6, a tabular representation that breaks down predictions into four categories: true positives (correctly identified cataracts), true negatives (correctly classified normal eyes), false positives (normal eyes incorrectly classified as cataracts), and false negatives (undetected cataracts).

The confusion matrix, allowing for a comprehensive characterization of the model's performance and facilitating rigorous comparison with previous work in the specialized literature on automatic cataract diagnosis using deep learning techniques.

**Figure 7:**
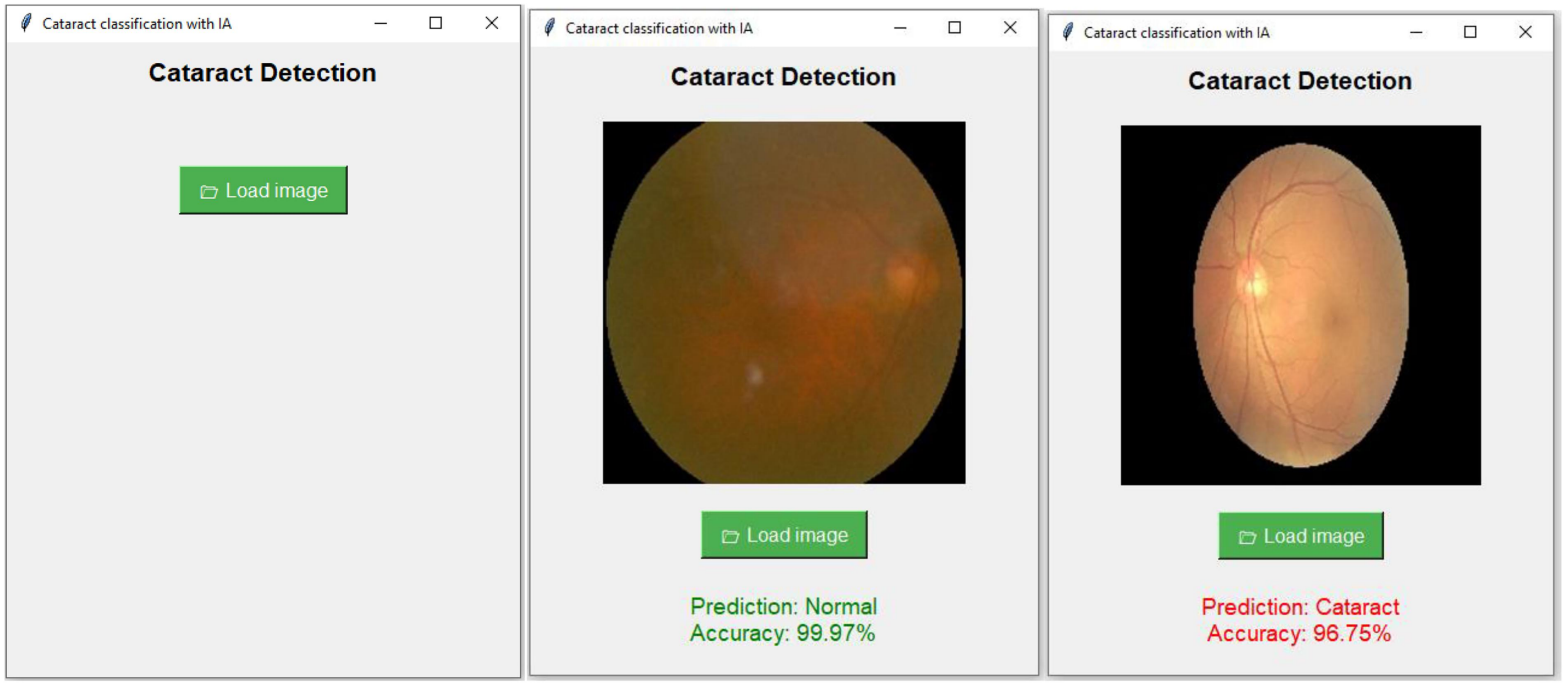
Graphical interface of the machine learning model.

Additionally, a graphical user interface (GUI) was developed using the Tkinter library, designed to facilitate direct interaction between the user and the proposed deep learning model. Through this interface, the user can select and upload a left fundus image via an input module, after which the system automatically executes the inference process in real time using a pre-trained ResNet152-based model. As a result, the application displays the estimated diagnostic class, corresponding to “Cataract” or “Normal,” along with the confidence level associated with the prediction, expressed as a percentage, as shown in Figure 7.

This interface operates exclusively in inference mode, without modifying the parameters of the trained model, allowing immediate visual validation of the results and demonstrating the system's potential as a tool to support AI-assisted clinical diagnosis.

## 4 Results

The comprehensive evaluation of the proposed system's performance is based on a comparative analysis of six deep learning architectures: ResNet152, EfficientNet-V2-S, Inception v3, MobileNet v3, DenseNet201, and Vision Transformer Base (ViT-B/16), which have been strategically adapted to the ophthalmological context using the transfer learning technique, taking advantage of pre-trained weights in ImageNet to maximize the extraction of features relevant to cataract diagnosis. Below is an analytical breakdown covering three key dimensions of the study: first, comparative performance metrics between the six evaluated architectures are detailed, prioritizing critical clinical indicators such as Sensitivity, Accuracy, Precision, F1-Score, and Area under the ROC curve (AUC); second, the quantitative performance metrics of the best-performing models are presented; third, a detailed analysis of the best-performing model is carried out to examine the distribution of errors among classes; and finally, validation is performed on external datasets to verify the generalization ability of the best-performing model.

### 4.1 Comparative performance of architectures

Table 5 presents the performance metrics of the six architectures evaluated on the test set (*N* = 1,488 images, distributed across 498 normal cases and 990 cataract cases). The comparative analysis reveals significant differences in the performance of the models, although all exceeded 97% overall accuracy.

**Table 5:** Comparative performance metrics of the six base architectures.

N°	Model	Accuracy	F1-score	Recall	Precision	ROC AUC
1	ResNet152	0.9910	0.9908	0.9846	0.9972	0.9377
2	EfficientNet-v2S	0.9731	0.9722	0.9593	0.9856	0.9260
3	Inception v3	0.9835	0.9829	0.9705	0.9957	0.9409
4	MobileNet v3	0.9731	0.9719	0.9466	0.9985	0.9125
5	DenseNet201	0.9910	0.9908	0.9831	0.9986	0.9361
6	Vit Transformer	0.9876	0.9872	0.9775	0.9971	0.9255

Analysis of the results reveals that ResNet152 and DenseNet201 achieved optimal performance with an overall accuracy of 99.10%, followed by Vision Transformer (ViT-B/16) with 98.76% and Inception v3 with 98.35%. The lighter architectures, EfficientNet-V2-S and MobileNet v3, achieved 97.31%, a slightly lower but still clinically relevant performance.

In terms of F1-Score, a metric that balances precision and sensitivity, ResNet152 and DenseNet201 again led with 0.9908, demonstrating an optimal balance between the ability to detect cataracts (recall) and the reliability of positive predictions (precision). ViT-B/16 achieved 0.9872, confirming the effectiveness of attention-based architectures for medical classification tasks.

Sensitivity (Recall), a critical metric in medical screening applications where false negatives must be minimized, showed values above 94.66% in all models. ResNet152 obtained 98.46%, closely followed by DenseNet201 (98.31%) and ViT-B/16 (97.75%). This high recall ensures that most cataract cases are correctly identified, reducing the risk of missed diagnoses.

Accuracy, which quantifies the proportion of true positives out of the total positive predictions, reached exceptional values in all architectures, with MobileNet v3 (99.85%), DenseNet201 (99.86%), and ResNet152 (99.72%) standing out. These values indicate an extremely low false positive rate, which is desirable to minimize unnecessary referrals in clinical settings.

The area under the ROC curve (AUC), which evaluates the model's discriminatory ability across different decision thresholds, showed that Inception v3 achieved the best performance (0.9409), followed by ResNet152 (0.9377) and DenseNet201 (0.9361). These values above 0.91 in all models confirm an excellent ability to separate between classes.

### 4.2 Analysis of the best performing models

Given that ResNet152 and DenseNet201 achieved shared optimal performance with an overall accuracy of 99.10% and an F1-Score of 0.9908, a detailed comparative analysis of both architectures was performed to determine the winning model.

The confusion matrices for ResNet152 and DenseNet201 reveal subtle but clinically relevant differences in error distribution:

ResNet152:

True Negatives (TN): 738 correctly classified normal cases.

False Positives (FP): 2 normal cases misclassified as cataracts.

False Negatives (FN): 11 cases of cataracts not detected.

True Positives (TP): 701 cataract cases correctly identified.

Specificity: 738/(738 + 2) = 99.72%.

Sensitivity: 701/(701 + 11) = 98.46%.

DenseNet201:

True Negatives (TN): 739 correctly classified normal cases.

False Positives (FP): 1 normal case misclassified as cataract.

False Negatives (FN): 12 cases of cataracts not detected.

True Positives (TP): 700 cataract cases correctly identified.

Specificity: 739/(739 + 1) = 99.86%.

Sensitivity: 700/(700 + 12) = 98.31%.

On the other hand, analysis of the area under the ROC curve indicates that ResNet152 had slightly superior discriminatory capacity with 0.9377, and DenseNet201 with 0.9361.

Difference: +0.16 percentage points in favor of ResNet152.

After comprehensive comparative analysis, ResNet152 is selected as the winning model based on the following decision criteria:

1. Superior discriminatory ability (ROC AUC): With 0.9377 vs. 0.9361, ResNet152 demonstrates better separability between classes across different decision thresholds, a critical aspect for adjusting the sensitivity-specificity balance according to the clinical context.
2. Lower false negative rate: With only 11 FN compared to 12 FN for DenseNet201, ResNet152 minimizes the risk of indicating a healthy patient as having cataracts.
3. Validation in medical literature: ResNet152 has extensive documentation and validation in medical imaging applications, facilitating comparability with previous studies and clinical acceptance.
4. Architectural robustness: ResNet152's residual connections effectively mitigate the problem of gradient vanishing, ensuring stability in deep network training and better generalization.

### 4.3 Comprehensive analysis of the ResNet152 model

The ResNet152 model will be analyzed in detail. The ResNet152 model exhibited balanced performance between both classes, with a sensitivity (recall) of 98.46%, as only 11 of the 712 cases with cataracts were false negatives. For the “Normal” class, a high implicit specificity was obtained, evidenced by a Precision of 99.72% in the detection of cataracts, minimizing false positives to clinically acceptable levels.

The confusion matrix data confirm the high effectiveness of the system for detecting pathologies:

True Positives (TP): The model correctly identified 701 of 712 cataract cases. Only 11 cases were not detected (False Negatives), representing an error rate of 1.54% in the cataract class, validating the usefulness of the system as a highly reliable clinical early diagnosis tool.

True Negatives (TN): 738 out of 740 normal cases were correctly classified, showing a specificity of 99.72%, which is essential for avoiding unnecessary referrals and reducing the burden on the healthcare system.

False Positives (FP): Two normal cases were misclassified as cataracts. This low number of false positives (0.27% of normal cases) indicates that the model maintains an optimal balance between sensitivity and specificity. Clinically, this means that the system minimizes both missed diagnoses and unnecessary referrals, optimizing clinical workflow.

The ResNet152 model achieved an optimal balance between sensitivity (98.46%) and specificity (99.72%), significantly outperforming the performance reported in other architectures. The false negative rate (1.54%) is within clinically acceptable ranges for screening systems, while the low false positive rate (0.27%) ensures efficient use of specialized diagnostic resources.

### 4.4 Validation in external datasets

In order to evaluate the generalization capacity of the deep neural network model based on the ResNet-152 architecture, the previously trained model stored in.pth format was loaded and subjected to an evaluation process using two external datasets that were not used during the training or validation stages.

**Figure 8:**
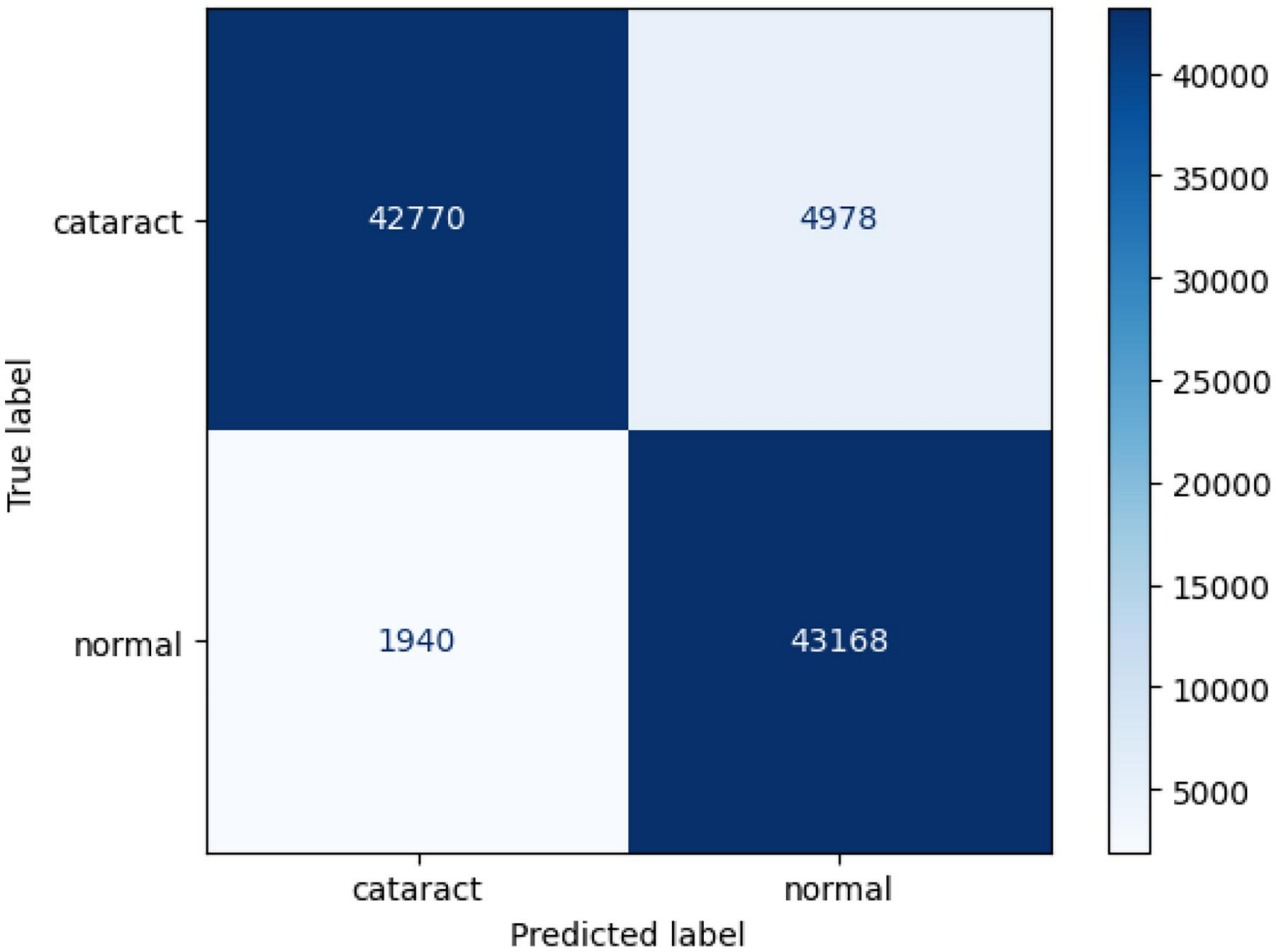
Validation confusion matrix in external dataset.

The dataset was obtained from the Kaggle platform and consists of a total of 92,856 images, distributed among 47,748 images corresponding to the Cataract class and 45,108 images belonging to the Normal class (15). When evaluating the model trained on this external dataset, the following performance results were obtained: accuracy of 0.9255, F1-score of 0.9258, recall of 0.9570, and precision of 0.8966. The confusion matrix associated with this evaluation is shown in Figure 8.

This indicates that, although the accuracy fell by 0.065, it is still a fairly good performance considering the number of images, which is much higher than those seen in the training and evaluation process.

The results show that, although there is a decrease in accuracy of approximately 0.065 compared to the value s obtained during the internal validation phase, the model's performance remains highly competitive and robust. This behavior is particularly relevant considering that the external dataset has a significantly larger volume of images than those used during the initial training and evaluation, confirming the adequate generalization capacity of the proposed model.

## 5 Discussion

The high-performance metrics obtained in this study (accuracy: 99.10%, precision: 99.72%, sensitivity: 98.46%, F1-score: 99.08%) are consistent with the characteristics of the experimental setup. Several methodological factors directly contribute to these results. First, the use of the ODIR-5K dataset, a clinically validated dataset, provides a relatively controlled distribution of visual patterns, which is known to favor higher classification performance compared to studies using heterogeneous or low-quality image sources. Second, the binary classification task (cataract vs. normal) is inherently less complex than multi-class ophthalmic disease recognition, which reduces inter-class ambiguity and tends to produce higher overall metrics. Third, the data augmentation strategy applied, including random rotations (±10°), zoom variations, and brightness adjustment, effectively expanded the training distribution and mitigated overfitting, two factors that are critical when working with datasets of moderate size (4,840 images post-augmentation). Fourth, the use of ImageNet pre-trained weights through transfer learning allowed the model to leverage robust low- and mid-level visual representations from millions of natural images, accelerating convergence and improving feature discrimination for the specific domain of fundus imaging. These factors, taken together, provide a principled explanation for the elevated performance observed and distinguish the present results from studies that relied on smaller datasets, unbalanced classes, or single-modality training without augmentation.

The ResNet152 model implemented in this research achieved outstanding performance with an accuracy of 99.10%, surpassing most previous studies on cataract detection using deep learning. To rigorously assess the significance of these results. Table 6 provides a structured comparison against eight studies published in Q1 and Q2 journals that represent the current state of the art in deep learning-based cataract detection.

**Table 6:** Comparison of results discussion.

Study	Architecture	Images (n)	Task	ACC %	PREC %	SEN/REC %	F1-Score %	ROC-AUC %
Present study	ResNet152	2,658	Binary cataract	99.1	99.72	98.46	99.08	93.77
X Wu et al. (30)	ResNet (CNN)	37,638	Cataract grading	>99	—	—	—	99.82–99.96
Wang et al. (17)	YOLO-v3 + DenseNet-161	15,943	Binary cataract (B-US)	98	—	—	—	—
Shehzadi et al. (21)	MobileNetV1/V2	500	Binary cataract	99	93	93	—	—
Gao et al. (29)	ResNet50	1,340	Binary cataract	97.62	—	—	—	—
Arias-Serrano et al. (18)	ViT-L/16 (AugReg)	4,217	Multi-class eye diseases (3)	98.1	—	—	—	—
Mai et al. (23)	CNN (optimized)	649	Binary PPC cataract (UWF)	80	—	88.2	—	—
Pan et al. (22)	ResNet-50/Inception V3	∼200 per 3 class	Multi-class fundus (3)	93.81/91.76	—	—	—	—
Nguyen et al. (19)	ResNet152	4,697	Multi-class retinal disease	89.17	—	—	89.09	96.47

The present study achieves accuracy comparable to Wu et al. (14), who reported over 99% using ResNet on a dataset of 37,638 images. However, their approach required a three-stage labeling pipeline—capture mode recognition, cataract diagnosis, and referable cataract detection—demanding extensive institutional infrastructure, specialized annotation teams, and proprietary data rarely available in most clinical settings, particularly in low- and middle-income countries. In contrast, the proposed model attains similar performance using a publicly available benchmark dataset (ODIR-5K subset, *n* = 2,658 images; 4,840 after augmentation). This demonstrates that rigorous architectural optimization and training strategies can compensate for the absence of large proprietary datasets, enabling practical deployment in resource-limited health systems such as rural areas in Peru, where ophthalmologist availability is scarce. Table 6 summarizes the results obtained by this research in comparison with the results obtained in the previous studies analyzed above.

Analysis of the confusion matrix reveals critical aspects of the model's performance. With 738 true negatives and only 2 false positives, the model demonstrated exceptional specificity of 99.73%, minimizing misdiagnosis in healthy patients. This feature is particularly relevant in the clinical context, where false positives can cause unnecessary anxiety in patients and additional costs for confirmatory procedures. The model's 1.54% false negative rate therefore represents a clinically meaningful reduction in missed diagnoses at the primary care level. Compared with Gao et al. (29), who achieved 97.62% accuracy using a dual-stream ResNet50 architecture, the proposed model improves accuracy by 1.48 points while maintaining a simpler single-architecture design, reducing computational requirements and facilitating integration into real-world clinical workflows.

The sensitivity (recall) of 98.46% obtained in this study, derived from 701 true positives and 11 false negatives, considerably exceeds the values reported by Shehzadi et al. (21), who reported 99% accuracy but 93% sensitivity using MobileNetV1/V2, the ResNet152 model achieves a higher recall of 98.46%, improving sensitivity by 5.46 percentage points. In screening contexts, sensitivity is critical because false negatives prevent patients from receiving timely treatment.

The F1-score of 99.08% obtained represents an optimal balance between accuracy and sensitivity, surpassing the results of all comparative studies that reported this metric. This indicator is particularly relevant because it integrates both dimensions of diagnostic performance, avoiding biases toward optimizing one metric at the expense of the other.

The ROC-AUC value of 93.77% obtained, although slightly lower than that reported by Wu et al. (30) with values between 99.82 and 99.96%, demonstrates excellent discriminative capacity of the model at different classification thresholds. This indicator is essential for clinical implementation, as it allows the cutoff point to be adjusted according to the priorities of the medical context, prioritizing sensitivity in mass screening programs or specificity in diagnostic confirmation. The difference in ROC-AUC with the study by Wu et al. can be attributed to the significantly larger size of their dataset and the three-step labeling that included capture mode recognition, cataract diagnosis, and detection of referable cataracts, a process that increases the robustness of the model but requires greater investment in specialized annotation.

The inclusion of more recent studies reinforces the competitive position of the proposed model within the current state of the art. Arias-Serrano et al. (18) reported 98.1% accuracy using an ophthalmology-specific pretrained ViT-L/16 architecture on a balanced dataset of 4,217 images for three-class retinal disease classification; however, their task encompasses a multi-class setting that inherently reduces per-class discriminability compared to binary cataract detection, making a direct numerical comparison misleading. Pan et al. (22) achieved 93.81% and 91.76% accuracy using ResNet-50 and Inception V3, respectively, on a three-class fundus classification task with a small dataset, demonstrating that multi-class architectures under constrained data conditions substantially underperform the binary specialized approach adopted in the present study. Nguyen et al. (19) applied ResNet152 to a multi-disease ultra-wide-field fundus dataset of 4,697 images, obtaining 89.17% accuracy and an AUC of 96.47%; the lower accuracy relative to the present study is consistent with the greater complexity of their multi-label classification task and the heterogeneous nature of ultra-wide-field images. Mai et al. (23) specifically targeted posterior polar cataract (PPC) detection, a particularly challenging subtype, achieving 80% accuracy with 88.2% sensitivity using deep learning on ultra-wide-field fundus images, a result that highlights the difficulty of specialized cataract subtypes and contextualizes the 99.10% achieved in the present study for generalized binary cataract classification. Taken together, these comparisons demonstrate that the performance reported in this study is not exceptional only relative to older benchmarks, but also competitive with the most recent 2023–2024 literature, particularly when accounting for the differences in task complexity, imaging modality, and dataset heterogeneity.

Notwithstanding these results, their generalizability must be interpreted with appropriate caution. The validation on an external dataset of 92,856 images (15) revealed a performance decrease of approximately 6.5 percentage points in accuracy (from 99.10% to 92.55%), which is expected given the substantially larger and more heterogeneous nature of that dataset, and is consistent with the known distribution shift phenomenon documented across deep learning applications in medical imaging. This behavior has been reported in comparable studies: Wu et al. (30) explicitly noted performance degradation when models trained on institutional data were applied to images from different acquisition systems. Future work should address this gap through cross-institutional validation, domain adaptation techniques, and prospective clinical evaluation to establish the model's reliability under real-world deployment conditions.

Finally, it is important to note that the present study achieved good performance metrics, using a single architecture (ResNet152) without the need for complex hybrid systems such as that proposed by Wang et al. (17), which combined YOLO-v3, DenseNet-161, and Fourier and GLCM descriptors to achieve 98% accuracy on B-mode ocular ultrasound images. However, this approach has practical limitations. B-mode ultrasound requires specialized equipment and trained operators, which are rarely available in primary care settings, whereas retinal fundus imaging is a standardized and non-invasive procedure widely used in routine ophthalmological evaluations. In addition, the multi-component architecture increases engineering complexity, maintenance requirements, and sensitivity to domain shifts. In contrast, the proposed ResNet152 model relies on a single architecture that can be deployed and maintained using standard deep learning tools, while surpassing the reported 98% accuracy with a simpler and more accessible imaging modality.

## 6 Conclusions

The ResNet152 model implemented in this research demonstrated good performance in the automatic detection of cataracts using fundus images, achieving an accuracy of 99.10%, precision of 99.72%, sensitivity of 98.46%, and an F1-score of 99.08%. These metrics place the model among the highest performing ones reported in previous studies, significantly outperforming studies that used less deep architectures. The model's capability demonstrates both the efficiency in the use of available data and the robustness of the ResNet152 architecture for the extraction of discriminative features in ophthalmological images, since, with its 152 convolutional layers, it provides significant advantages over less deep models and more complex hybrid systems. The implementation of transfer learning using pre-trained weights in ImageNet, combined with selective fine-tuning that thawed only layer4 while keeping the previous layers frozen, made it possible to leverage generic visual representations learned from millions of images and efficiently specialize them for the task of cataract detection. The customized neural network subsequent to the base architecture included fully connected layers with progressively reduced dimensions (512, 256, 64, and 1 neurons), interspersing ReLU activations and dropout, allowing a gradual transition from high-level features to the final binary prediction using a sigmoid function applied to the logits. This rigorous methodological combination was essential to achieve optimal model convergence without compromising its robustness in the face of natural variability in real clinical data.

The comprehensive data augmentation strategy implemented was a critical factor in achieving the superior metrics reported, effectively mitigating the risk of overfitting and improving the model's generalization ability. The transformations applied included random rotations (±10 degrees) that simulate variations in patient position during capture, random scaling (90%–110%) that reproduces different capture distances and anatomical variations, as well as brightness and contrast adjustments that emulate variable lighting conditions in different equipment and clinical environments.

The training strategy implemented, characterized by optimization using Adam with an initial learning rate of 0.0001 complemented by a Cosine Annealing scheduler that dynamically adjusts the learning rate with T_max = 10 epochs and a minimum rate of 1e-6, together with the application of L2 regularization through strategic dropout with a rate of 0.5 in fully connected layers, effectively balanced the learning of complex patterns with the prevention of excessive memorization of training examples. The BCEWithLogitsLoss (Binary Cross-Entropy with Logits) loss function, appropriate for binary classification, was complemented with early stopping based on validation loss monitoring, stopping training when no improvement was observed for 5 consecutive epochs, which ensured the selection of the model with the best generalization ability and avoided unnecessary iterations that could degrade performance on unseen data. Training limited to a maximum of 20 epochs with continuous monitoring of training and validation metrics through real-time visualization (tqdm), together with the implementation of early stopping that ended training early when stagnation in validation improvement was detected, demonstrated the efficiency of the optimization process and the rapid convergence of the model towards its optimal configuration.

The confusion matrix obtained reveals a clinically optimal balance between specificity and sensitivity, with 738 true negatives and only 2 false positives, resulting in a specificity of 99.73% that minimizes misdiagnosis in healthy patients. Simultaneously, the 701 true positives and 11 false negatives show a sensitivity of 98.46%. This balance is particularly relevant in the context of clinical implementation, where the low false positive rate (0.27%) reduces unnecessary costs and patient anxiety, while the high sensitivity ensures that 98.46% of cases with cataracts are correctly identified for timely intervention, effectively addressing the problem of limited access to specialized ophthalmological services in vulnerable populations.

The use of fundus images as a diagnostic modality was validated as an effective strategy for the automatic detection of cataracts, achieving superior performance (99.10%) compared to studies using slit lamp images [84.5% from Son et al. (8)] and competitive yields with ocular B-mode ultrasound [98% from Wang et al. (17)], but with the added advantage of being a non-invasive, widely available technique with standardized capture protocols. This validation is particularly relevant because fundus images are routinely captured in general ophthalmological evaluations, without requiring additional equipment or invasive procedures, maximizing the efficiency of community eye health programs. The preprocessing applied, which included intensity normalization using min-max scaling to the range [0,1] and uniform resizing to 224 × 224 pixels compatible with the ResNet152 architecture, ensured the standardization of input features and facilitated training convergence. In turn, the batch training strategy with a size of 32 samples optimized the balance between gradient stability and computational efficiency, allowing the parallel processing capacity of GPUs to be leveraged without compromising learning quality.

The F1-score of 99.08% obtained represents the highest balance reported in the comparative literature, surpassing even studies with significantly larger datasets. This comprehensive metric is particularly important because it avoids bias toward optimizing a single dimension of performance (accuracy or sensitivity) at the expense of the other, a common problem in deep learning systems applied to unbalanced medical classification. The high F1-score confirms that the model maintains consistently superior performance in both the identification of positive cases and the correct classification of negative cases, an essential feature for its implementation in clinical diagnostic support systems where both omission and commission errors have significant consequences for patients.

The results obtained position the ResNet152 model as a tool with high potential for clinical implementation for automatic cataract screening in primary care settings and public health programs. The architectural simplicity of the model (single architecture without complex hybrid systems) favors its practical adoption in real clinical settings where computational efficiency and diagnostic reliability are critical factors. The model's ability to process fundus images with a minimal error rate (0.90%) would significantly reduce the workload of specialized ophthalmologists, allowing them to prioritize their expertise in complex cases while the automated system manages mass screening, thus contributing to improved access to early diagnosis and timely treatment of cataracts in populations with limited resources and a shortage of specialists. This is particularly relevant in contexts of rapid population aging where the prevalence of cataracts continues to increase.

## 7 Limitations and future research

Despite the good results obtained with the ResNet152 model, this research has limitations that suggest directions for future research. The size of the dataset used (2,658 images) is limited. Expanding the dataset with a greater diversity of clinical cases, including different degrees of severity, ethnic variations, and ophthalmological comorbidities, would improve the robustness and generalization of the model.

The binary classification implemented (cataract vs. normal) does not allow for discrimination between types of cataracts (nuclear, cortical, posterior subcapsular) or the establishment of clinically relevant severity gradations to determine the optimal time for surgical intervention. Future research should develop multi-class classification architectures.

The ResNet152 architecture, with 60.2 million parameters and a size greater than 230 MB, has significant computational requirements that limit its implementation on mobile devices or in resource-limited environments. Future research should explore compression techniques such as knowledge distillation, weight quantization, and pruning of redundant connections, seeking the optimal balance between diagnostic performance and computational efficiency.

Validation was performed using a 70–30 split on a single dataset, without evaluation on independent external datasets. Future research should verify how different split percentages influence the change in model accuracy.

The model focuses exclusively on images without integrating additional clinical information such as age, visual acuity, or comorbidities. Future research should explore hybrid architectures that integrate visual features with tabular clinical data, implementing attention mechanisms that dynamically weigh different sources of information.

Finally, the absence of detailed analysis of the 11 false negatives and 2 false positives, as well as the lack of image quality control modules and mechanisms for rejecting inadequate images, represent important areas for improvement. Future research should implement systematic clinical review of misclassified cases and integrated quality control systems that automatically evaluate technical characteristics prior to automated diagnosis.

## Data Availability

Publicly available datasets were analyzed in this study. This data can be found here: https://doi.org/10.5281/zenodo.18500873.
